# RNA Sequencing Analysis Reveals Interactions between Breast Cancer or Melanoma Cells and the Tissue Microenvironment during Brain Metastasis

**DOI:** 10.1155/2017/8032910

**Published:** 2017-01-22

**Authors:** Ryo Sato, Teppei Nakano, Mari Hosonaga, Oltea Sampetrean, Ritsuko Harigai, Takashi Sasaki, Ikuko Koya, Hideyuki Okano, Jun Kudoh, Hideyuki Saya, Yoshimi Arima

**Affiliations:** ^1^Division of Gene Regulation, Institute for Advanced Medical Research, Keio University School of Medicine, Tokyo, Japan; ^2^Department of Respiratory Medicine, Kumamoto University, Kumamoto, Japan; ^3^Department of Breast Surgical Oncology, Tokyo Medical University, Tokyo, Japan; ^4^The Center for Integrated Medical Research, Keio University School of Medicine, Tokyo, Japan; ^5^Department of Physiology, Keio University School of Medicine, Tokyo, Japan; ^6^Laboratory of Gene Medicine, Keio University School of Medicine, Tokyo, Japan

## Abstract

Metastasis is the main cause of treatment failure and death in cancer patients. Metastasis of tumor cells to the brain occurs frequently in individuals with breast cancer, non–small cell lung cancer, or melanoma. Despite recent advances in our understanding of the causes and in the treatment of primary tumors, the biological and molecular mechanisms underlying the metastasis of cancer cells to the brain have remained unclear. Metastasizing cancer cells interact with their microenvironment in the brain to establish metastases. We have now developed mouse models of brain metastasis based on intracardiac injection of human breast cancer or melanoma cell lines, and we have performed RNA sequencing analysis to identify genes in mouse brain tissue and the human cancer cells whose expression is associated specifically with metastasis. We found that the expressions of the mouse genes* Tph2*,* Sspo*,* Ptprq*, and* Pole* as well as those of the human genes* CXCR4*,* PLLP*,* TNFSF4*,* VCAM1*,* SLC8A2*, and* SLC7A11* were upregulated in brain tissue harboring metastases. Further characterization of such genes that contribute to the establishment of brain metastases may provide a basis for the development of new therapeutic strategies and consequent improvement in the prognosis of cancer patients.

## 1. Introduction

Metastasis of cancer cells to the brain occurs in 9% to 17% of cancer patients, with the major sources of these cells being lung adenocarcinoma, breast cancer, and melanoma [1]. As a result of recent advances in systemic treatment of primary tumors, individuals with cancer are living longer and the incidence of brain metastasis is expected to increase. In addition to surgery, radiation, and cytotoxic chemotherapy, molecularly targeted therapies have recently been added to the treatment options for metastatic brain tumors and have improved outcome [2]. Despite the progress in multimodal treatment for brain metastases, however, the prognosis for affected patients remains poor [3]. For patients with human epidermal growth factor receptor 2– (HER2–) positive breast cancer or epidermal growth factor receptor mutation–positive non–small cell lung cancer, the brain remains a frequent site of disease recurrence regardless of disease control for primary tumors by systemic treatment with molecularly targeted agents such as trastuzumab or gefitinib, respectively [4, 5].

Several comprehensive analyses of gene expression signatures associated with brain metastasis have been performed for both clinical brain metastases and experimental brain metastasis models in order to provide insight into the molecular mechanisms underlying this process. Such analyses of human brain metastasis have contributed to the identification of predictive markers as well as providing a basis for the development of novel therapeutic targets [6, 7]. Analysis of mouse experimental models has identified several genes that mediate the metastasis of breast cancer and melanoma cells to the brain [8, 9]. Extracellular vesicles, or exosomes, released by cancer cells have also been found to promote metastasis in an organ-specific manner [10, 11]. These and other studies have searched for molecules associated with brain metastasis by focusing in large part on the metastatic cancer cells. However, given that the tumor microenvironment (TME) is also now thought to play a key role in metastasis [12, 13], it is imperative to investigate simultaneously the signaling pathways that support metastasis in both cancer cells and stromal cells of the TME.

Metastatic colonization—the outgrowth of cancer cells in distant organs—is the most complex and rate-limiting phase of metastasis, with cross talk between cancer cells and the TME being an important determinant of this process [14, 15]. Niche interactions mediated by E-cadherin and N-cadherin promote bone colonization by breast cancer cells [16], and signaling mediated by the chemokine CXCL12 and its receptor CXCR4 facilitates the recruitment of CXCR4^+^ cancer cells to bone [17, 18]. Lysyl oxidase secreted by breast cancer cells has also been found to influence bone homeostasis by modulating osteoclastogenesis driven by the transcription factor NFATc1, thereby contributing to the establishment of a platform that supports the colonization of circulating tumor cells and subsequent formation of bone metastases [19]. Cancer cells and stromal cells thus cooperate in the development of metastatic lesions, and the identification of molecular interactions related to metastasis will require an understanding of the roles played by both cell types.

Several approaches that take advantage of the species difference in xenograft tumor models to acquire gene expression profiles in both cancer cells and stromal cells simultaneously have recently been developed [20–22]. We have now established xenograft models of brain metastasis and performed RNA sequencing analysis of metastatic lesions in these models. Separate analysis of the transcriptomes of cancer cells and the TME revealed candidate genes in both associated with brain metastasis. Our results suggest that this approach is valuable for investigation of the cross talk between cancer cells and the TME during brain metastasis.

## 2. Materials and Methods

### 2.1. Cell Lines and Cell Culture

The human breast cancer cell line MDA-MB-231-Luc-D3H2LN (231-Luc) was obtained from Caliper Life Sciences (Hopkinton, MA, USA). HER2-expressing 231-Luc cells—HER2-60 and HER2-90 cells—were established as previously described [23]. These breast cancer cell lines were cultured in minimum essential medium/Earle's Balanced Salt Solutions supplemented with 10% FBS, nonessential amino acids, L-glutamine, sodium pyruvate, and minimum essential medium vitamin solution. Human breast cancer cell line HCC1937 cells were obtained from American Type Culture Collection (Manassas, VA, USA) and cultured in Dulbecco's modified Eagle's medium supplemented with 10% FBS. The human melanoma cell line MeWo was obtained from American Type Culture Collection (Manassas, VA, USA) and cultured in Eagle's minimum essential medium supplemented with 10% fetal bovine serum. Human melanoma WM3734 cells were obtained from Coriell Institute for Medical Research (Camden, NJ, USA) and cultured in 2% Tumor Medium (Tu2%), consisting of 80% MCD B153 medium and 20% Leibovitz's L-15 medium, supplemented with 2% fetal bovine serum, insulin (5 *μ*g/mL), and 1.68 mM CaCl_2_ [24]. All cells were maintained at 37°C under a humidified atmosphere of 5% CO_2_.

### 2.2. Cell Transplantation

For orthotopic xenograft models, 231-Luc cells (2 × 10^5^ in 30 *μ*L of phosphate-buffered saline [PBS]) or HCC1937 cells (1 × 10^7^ in 30 *μ*L of PBS) were injected into the number 4 mammary fat pads of 4-week-old female Balb/c nu/nu immune-deficient mice (Charles River, Burlington, MA, USA) that had been anesthetized by exposure to 1% to 3% isoflurane. MeWo cells (1 × 10^6^ in 50 *μ*L of PBS) were injected subcutaneously into the lower flanks of 4-week-old female Balb/c nu/nu immune-deficient mice anesthetized by intraperitoneal administration of Somnopentyl (Kyoritsu Seiyaku, Tokyo, Japan).

For metastasis models based on intracardiac injection of cancer cells, PBS or cancer cells (1 × 10^5^ in 100 *μ*L of PBS) were injected into the left ventricle of 4-week-old female Balb/c nu/nu mice anesthetized by inhalation of 1% to 3% isoflurane or intraperitoneal administration of Somnopentyl (Kyoritsu Seiyaku, Tokyo, Japan). The* luciferase*-expressing cell tumor formation was confirmed by bioluminescence imaging. At 3 to 4 or 5 to 7 weeks after cell injection, the brain was removed from the skull of anesthetized mice and cut into eight pieces with the use of Brain Matrices (ASI Instruments, Warren, MI, USA) to check for breast cancer cells or melanoma cells, respectively. Bone marrow was also isolated from both femurs and tibiae of the injected mice and was incubated twice for 30 min at 37°C with a mixture of collagenase and hyaluronidase (Stemcell Technologies, Vancouver, British Columbia, Canada). The dissociated cells were then collected by passage through of a 40 *μ*m Cell Strainer (Corning, Corning, New York, USA) followed by centrifugation and resuspension in a red blood cell lysis buffer.

### 2.3. RNA-Seq

Total RNA was isolated from brain, bone marrow cells, and primary fat pad tumors with the use of the TRIzol reagent (Life Technologies, Carlsbad, CA, USA) and was evaluated by determination of the RNA integrity number (RIN). RNA sequencing (RNA-seq) was performed as previously described [25]. The mRNA libraries were prepared according to the TruSeq RNA Sample Prep Kit protocol and sequenced with a Genome Analyzer IIx (Illumina, San Diego, California, USA). Mouse and human mRNA sequences were separated with the use of Xenome software [21]. The separated RNA-seq data were mapped to the corresponding human (GRCh37) and mouse (mm9) genomic DNA sequences with the use of TopHat software [26]. The mapped sequences were normalized by the trimmed mean of M values (TMM) and analyzed with FeatureCount [27] and edgeR [28] software.

### 2.4. RT and Real-Time PCR Analysis

Total RNA was subjected to reverse transcription (RT) with a SuperScript III First-Strand Synthesis Kit (Thermo Fisher Scientific, Waltham, Massachusetts, USA), and the resulting cDNA was subjected to real-time polymerase chain reaction (PCR) analysis with SYBR Premix ExTaq (Takara Bio, Shiga, Japan) and a Thermal Cycler Dice Real Time System (TB800, Takara Bio). The amplification protocol comprised 40 cycles of incubations at 95°C for 30 s and at 60°C for 30 s. PCR primer sequences (forward and reverse, resp.) were as follows: 5′-ACTAACATCAAATGGGGTGAGGCC-3′ and 5′-GGATGCATTGCTGACAATCTTGAGTGA-3′ for* Gapdh*; 5′-GTTTTCCCAAGAGATAGGCTTAG-3′ and 5′-GACGAAAGTAACCCTGCTCCATAC-3′ for* Tph2*; 5′-GGGAAGAGCGTTTGTATTCG-3′ and 5′-CCGTACTCAGAGTGTCTTGCTG-3′ for* Sspo*; 5′-CATTCAGATCGACTGGACCAT-3′ and 5′-AGGCCAACCCGTGAAGTTAC-3′ for* Ptprq*; 5′-TTTACTCTCACCATCCGCACTG-3′ and 5′-CAGTTTGATACAGGGCTTGTCTG-3′ for* Pole*; 5′-CAAAATCAAGTGGGGCGATGCTGGC-3′ and 5′-GGCATTGCTGATGATCTTGAGGCT-3′ for* GAPDH*; 5′-CCTTGGAGCCAAATTTAAAACC-3′ and 5′-CAGACTCAGTGGAAACAGATGAATG-3′ for* CXCR4*; 5′-TGCTCTGCGGCAGTTGAC-3′ and 5′-GAAGAAGGCACTCACTCCATAG-3′ for* PLLP*; 5′-AATGTGACCACTGACAATACCTC-3′ and 5′-CACCAGGATTTTGATGGATAAG-3′ for* TNFSF4*; 5′-AAAGGCCCAGTTGAAGGATG-3′ and 5′-ATAGAGCACGAGAAGCTCAGG-3′ for* VCAM1*; 5′-ATTGCCGTGCTGCTGTAC-3′ and 5′-GGCGAAGAGGATGTACAGG-3′ for* SLC8A2*; and 5′-CCTCGACAGTCTTTTGAATTTCC-3′ and 5′-AAACAAAGCTGGGATGAACAGTG-3′ for* SLC7A11*.

### 2.5. Statistical Analysis

Quantitative data are presented as means ± SD and were subjected to analysis of variance followed by Dunnett's test with the use of Prism v.6 software (GraphPad Software, San Diego, CA, USA). A *P* value of <0.05 was considered statistically significant.

## 3. Results

### 3.1. Experimental Models of Breast Cancer and Melanoma Cell Metastasis to the Brain

**Figure 1:**
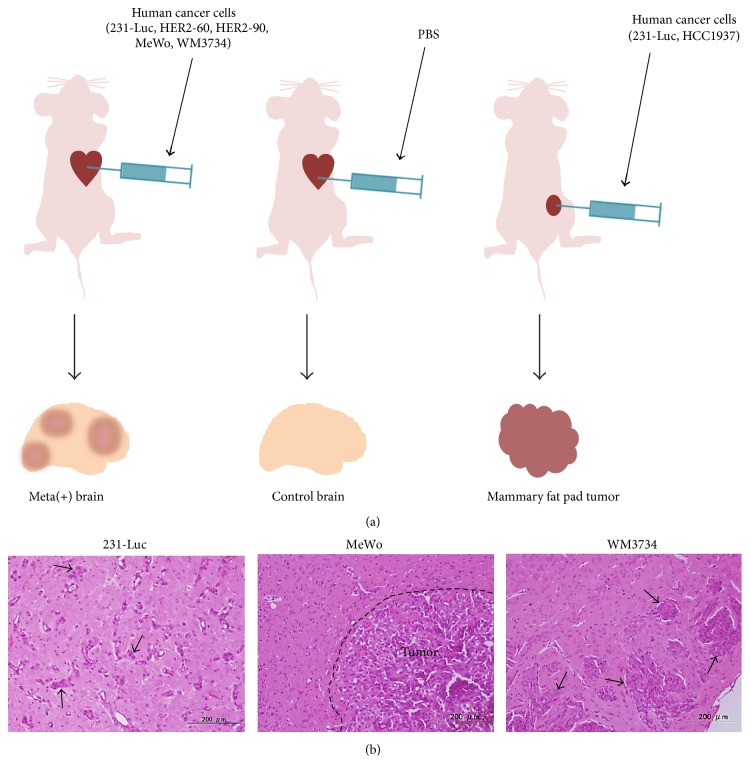
Xenograft models of brain metastasis and primary breast cancer. (a) For models of brain metastasis, human breast cancer (231-Luc, HER2-60, and HER2-90) or melanoma (MeWo, WM3734) cell lines (or PBS as a control) were injected into the left ventricle of female immunodeficient mice. For models of primary breast cancer, human 231-Luc or HCC1937 cells were orthotopically injected into mammary fat pads. (b) Hematoxylin-eosin staining of mouse brain tissue with metastases formed by 231-Luc, MeWo, or WM3734 cells. Arrows indicate human metastatic cancer cells. Scale bars, 200 *μ*m.

To develop xenograft models of brain metastasis, we injected human breast cancer cells (231-Luc, HER2-60, and HER2-90), human melanoma cells (MeWo, WM3734), or PBS into the heart of female nude mice (Figure 1(a)). We also injected 231-Luc or human breast cancer HCC1937 cells into mammary fat pads of nude mice as models of primary breast cancer (Figure 1(a)). Brain metastases were observed in the mice subjected to intracardiac injection of the breast cancer and melanoma cells (Figure 1(b)). The brain and bone marrow of the brain metastasis models as well as the primary fat pad tumors of the orthotopic breast cancer models were removed for isolation of total RNA. We confirmed that both human and mouse mRNAs were present in the isolated total RNA samples by RT and real-time PCR analysis with primers specific for the human or mouse glyceraldehyde-3-phosphate dehydrogenase (GAPDH) gene (data not shown). We also confirmed the quality of the RNA preparations by determining RIN values (data not shown).

### 3.2. Next-Generation Sequencing and Transcriptome Data Analysis

We performed RNA-seq analysis with a next-generation sequencer. The human and mouse RNA-seq data were separated with the use of Xenome software and were mapped to the human GRCh37 and mouse mm9 genomic DNA sequences with the use of TopHat software. These data were then analyzed with FeatureCount and edgeR software. Representative numbers of reads for RNA samples isolated from the brain and bone marrow of the metastasis model for 231-Luc cells as well as from primary fat pad tumors formed by these cells are shown in Table 1, with the human and mouse reads indicating transcripts derived from cancer cells and the TME, respectively.

### 3.3. Mouse Genes Related to Brain Metastasis

**Figure 2:**
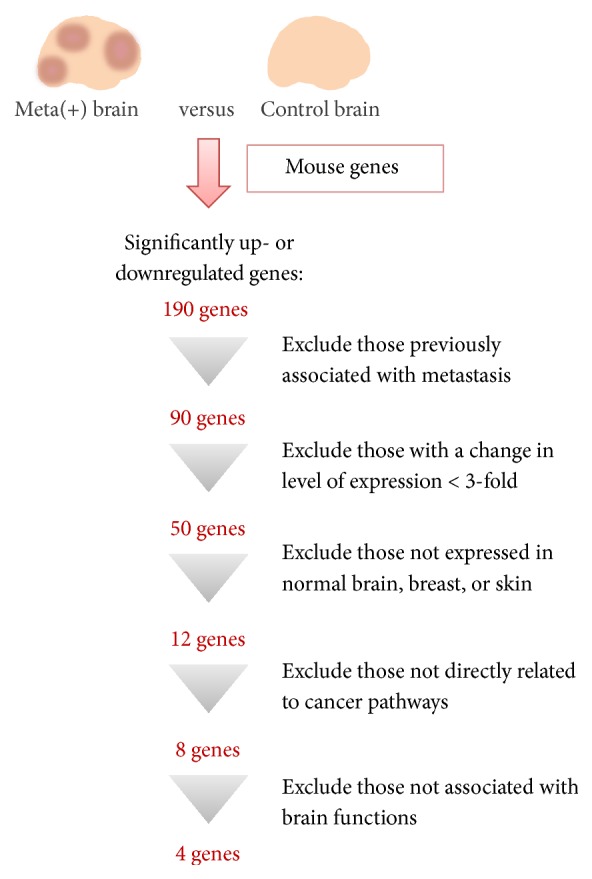
Flow chart for the identification of candidate genes whose expression in mouse brain cells is associated with metastasis of human breast cancer of melanoma cells. RNA-seq analysis was performed with control brain tissue or brain tissue harboring metastases formed by breast cancer cells (231-Luc, HER2-60, and HER2-90) or melanoma cells (MeWo, WM3734). Analysis of the mouse-specific transcriptome resulted in the identification of 190 genes whose expression was significantly up- or downregulated in brain tissue containing metastases [meta(+) brain]. Application of additional selection criteria winnowed this group of genes down to four.

**Figure 3:**
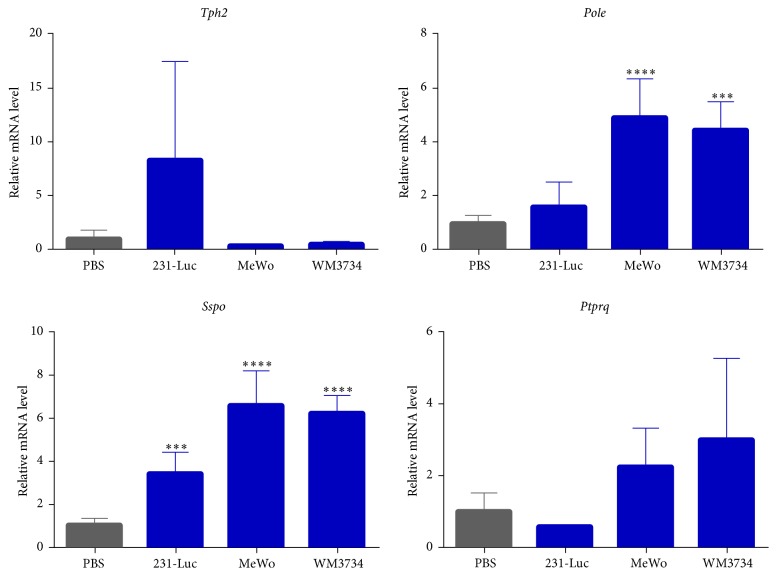
RT and real-time PCR analysis of candidate genes related to brain metastasis identified from the mouse transcriptome analysis. The abundance of* Tph2*,* Pole*,* Sspo*, and* Ptprq* mRNAs in the brain of mice with metastases formed by 231-Luc, MeWo, or WM3734 cells is presented relative to the corresponding value for the brain of mice injected with PBS. Data are means ± SD of triplicate determinations. ^*∗∗∗*^*P* < 0.001 and ^*∗∗∗∗*^*P* < 0.0001 versus the value for control (PBS-injected) mice.

To identify genes in the brain microenvironment whose expression is associated with metastasis, we compared the mouse-specific transcriptome of the control brain with that of brain tissue harboring metastases. Each anatomic site has unique combinations of gene expression patterns [29], so we cut the brains into eight pieces and analyzed the brain pieces from the same locations in both the control and the metastasis mice. The metastatic tumor samples comprised brain metastases formed by breast cancer cells (231-Luc, HER2-60, and HER2-90) or melanoma cells (MeWo, WM3734), whereas the control samples were from brains injected with PBS. After FeatureCount and edgeR analyses, we found 190 candidate genes that showed statistically significant differences between the meta(+) brains and the control brains. The expression of 190 mouse genes was significantly up- or downregulated in brain tissue with metastases (Supplemental Table  1 in Supplementary Material available online at https://doi.org/10.1155/2017/8032910). We then applied multiple selection criteria to identify novel candidate genes associated with brain metastasis (Figure 2). We first excluded 100 genes that had previously been associated with metastasis and then applied a cutoff for fold change in expression of >3. Exclusion of genes that are not expressed in normal brain, breast, or skin tissue followed by application of Ingenuity Pathway Analysis to exclude those that are not known to be directly related to cancer pathways resulted in the isolation of eight genes, only four of which—*Tph2*,* Sspo*,* Ptprq*, and* Pole*—were known to be associated with brain specific functions.* Tph2* encodes tryptophan hydroxylase 2, the rate-limiting enzyme in the synthesis of serotonin (5-hydroxytryptamine) [30, 31];* Sspo* encodes SCO-spondin, a member of the thrombospondin superfamily of proteins that is widely distributed in the central nervous system [32];* Ptprq* encodes protein tyrosine phosphatase receptor type Q, a receptor-like protein tyrosine phosphatase that also catalyzes the dephosphorylation of phosphatidylinositol 3,4,5-trisphosphate (PIP_3_); and* Pole* encodes the catalytic subunit of DNA polymerase *ε*, which participates in DNA repair and chromosomal DNA replication [33]. RT and real-time PCR analysis confirmed that the expression levels of mouse* Sspo* and* Pole* were significantly increased in brain tissue of metastasis model mice compared with control brain tissue (Figure 3).

### 3.4. Human Genes Related to Brain Metastasis

**Figure 4:**
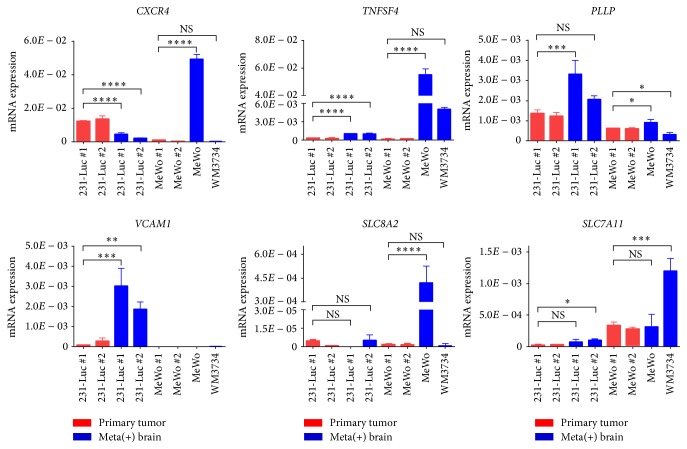
RT and real-time PCR analysis of candidate genes related to brain metastasis identified from the human transcriptome analysis. The abundance of* CXCR4*,* TNFSF4*,* PLLP*,* VCAM1*,* SLC8A2*, and* SLC7A11* mRNAs in the brain of individual mice (#1, #2) with metastases formed by 231-Luc, MeWo, or WM3734 cells, or in primary tumors formed by 231-Luc cells (mammary fat pad) or by MeWo cells (subcutaneous) in individual mice (#1, #2) were normalized by* GAPDH* mRNA. Data are means ± SD of triplicate determinations. ^*∗*^*P* < 0.05, ^*∗∗*^*P* < 0.01, ^*∗∗∗*^*P* < 0.001, and ^*∗∗∗∗*^*P* < 0.0001. NS: not significant.

To identify genes in cancer cells that contribute to metastasis, we analyzed the cancer cell–derived human transcriptome in our metastasis models. We compared the human-specific transcriptomes of brain or bone marrow from the metastasis models with those of corresponding primary tumor samples from the orthotopic breast cancer models. The primary tumor samples thus included fat pad tumors formed by 231-Luc or HCC1937 cells, whereas the metastatic tumor samples comprised brain metastases formed by breast cancer cells (231-Luc, HER2-60, and HER2-90) or melanoma cells (MeWo, WM3734) as well as bone metastases formed by breast cancer cells (231-Luc, HER2-60, and HER2-90) or melanoma cells (MeWo). We first compared the brain metastasis samples with the primary tumor samples, and then we compared the bone marrow metastasis samples with the primary tumor samples, and we tried to identify the genes commonly upregulated in both brain metastasis samples and the bone marrow metastasis samples, which were statistically significant. Although it was not sufficient to detect all annotated genes due to the lack of the number of reads that mapped to human genes in the metastatic tumor samples (1 to 5 million reads), we did find that the expressions of human* CXCR4*,* MIAT*,* PLLP*,* TNFSF4*,* VCAM1*,* SLC8A2*, and* SLC7A11* were upregulated in the metastasized cancer cells both in the brain and in the bone marrow. Given that* MIAT* encodes a noncoding RNA, we examined the expression levels of the remaining six genes by RT and real-time PCR analysis (Figure 4). Brain metastases formed by 231-Luc cells were compared with primary 231-Luc fad pad tumors, whereas those formed by MeWo or WM3734 cells were compared with primary MeWo skin tumors. The expression of the candidate genes was upregulated in the metastasized cancer cells.* CXCR4* encodes chemokine receptor 4, which is activated by binding of the chemokine CXCL12, with the CXCL12-CXCR4 signaling axis having been implicated in brain metastasis of breast cancer [34].* PLLP* encodes plasmolipin, a proteolipid protein found in kidney and brain.* TNFSF4* encodes tumor necrosis factor superfamily member 4 (also known as OX40L), which is the ligand for TNFRSF4 (OX40) and expressed on the surface of antigen presenting cells.* VCAM1* encodes vascular cell adhesion molecule 1, which is expressed on the surface of endothelial cells and interacts with integrins on the surface of leukocytes to mediate both cell adhesion and signal transduction. VCAM1 is also expressed on cancer cells, with its expression on breast cancer cells having been found to confer a survival advantage as a result of interaction of the cells with macrophages and consequent activation of a VCAM1–Ezrin–PI 3-kinase–Akt signaling pathway [35].* SLC8A2* encodes a Na^+^-Ca^2+^ exchanger whose expression is restricted to the brain [36].* SLC7A11* encodes the xCT subunit of a cystine-glutamate transporter that increases intracellular glutathione levels to protect cells from oxidative stress [37]. The fact that the roles of* CXCR4* and* VCAM1* in metastasis are well established [38, 39] supports the notion that these six candidate genes contribute to metastasis of cancer cells to the brain.

### 3.5. Interaction Networks of the Candidate Metastasis-Related Genes

**Figure 5:**
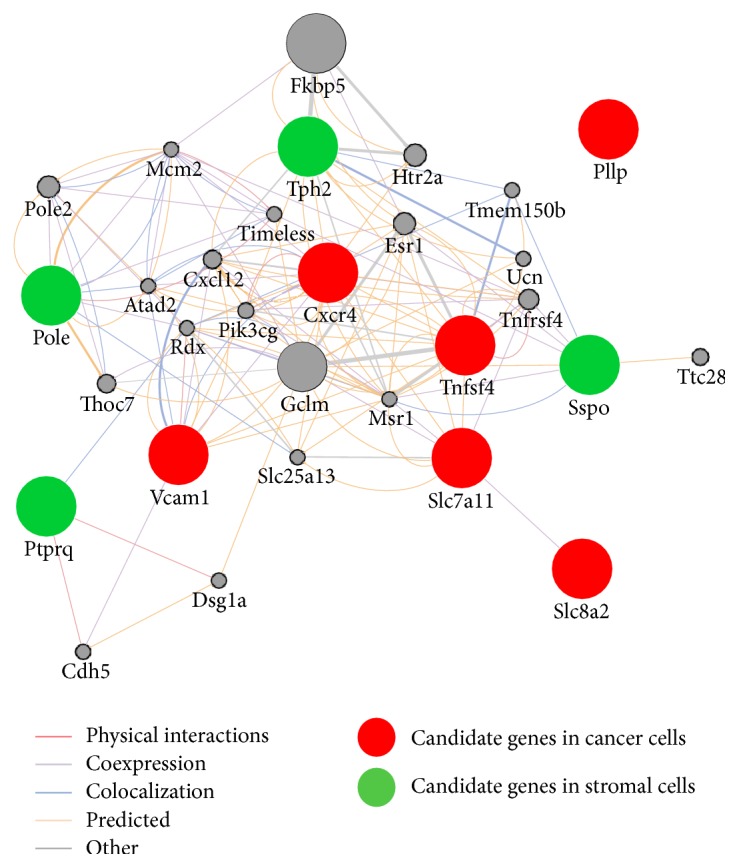
Functional interaction network analysis by GeneMANIA of candidate mouse and human genes related to brain metastasis. Constructed network pathway for the six and four genes identified in human cancer cells and mouse stromal cells, respectively. Up to 20 of the most related genes and 20 of the most related attributes are shown.

We analyzed the functional interaction networks of the genes implicated in brain metastasis in the present study with the use of the open-source platforms GeneMANIA (http://www.genemania.org) [40] and Cytoscape [41]. Analysis of the 10 candidate genes* (Tph2*,* Sspo*,* Ptprq*,* Pole*,* CXCR4*,* PLLP*,* TNFSF4*,* VCAM1*,* SLC8A2*, and* SLC7A11)* with GeneMANIA and Cytoscape revealed interactions among them (Figure 5), with the corresponding functions of these genes being predominantly related to immune responses (see the following list).

*Top 10 Functions from GeneMANIA*

T cell proliferation
Leukocyte proliferation
Lymphocyte proliferation
Mononuclear cell proliferation
Calcium ion transport
Activation-induced cell death of T cells
Regulation of immunoglobulin secretion
DNA polymerase complex
Branching morphogenesis of an epithelial tube
Positive regulation of cytosolic calcium ion concentration

## 4. Discussion

We have here identified four genes—*Tph2*,* Sspo*,* Pole*, and* Ptprq*—whose expression in the brain microenvironment was associated with metastasis of cancer cells. Cancer cells and the TME have been shown to interact functionally during metastatic colonization, with such interactions being considered as potential therapeutic targets [42]. Astrocytes in the brain play a key role in brain metastasis of lung and breast cancer cells. Direct contact with astrocytes thus increases the expression of survival genes in cancer cells [43] as well as supporting tumor growth and resistance to chemotherapy [44]. On the other hand, the production of plasminogen activator by astrocytes generates plasmin and thereby promotes Fas ligand–induced apoptosis in cancer cells and inhibits cancer cell growth in the brain, although some cancer cells express serpin proteins that inhibit plasminogen activator and thereby promote cancer cell survival [45].

According to the transcriptome database of mouse brain cells [46], TPH2, the rate-limiting enzyme in serotonin synthesis, is highly expressed in astrocytes. Serotonin is synthesized from tryptophan and functions as a neurotransmitter in the central nervous system, and it has been linked with various cancers [47]. Given that serotonin promotes the proliferation and survival of breast cancer cells [48], the upregulation of TPH2 in astrocytes might contribute to cancer cell growth in brain tissue through increased serotonin production.

PTPRQ is a member of the type III receptor-like protein tyrosine phosphatase family and plays a role in the regulation of cell proliferation and differentiation [49]. It inhibits PIP_3_-dependent signaling and thereby attenuates Akt activation as well as the proliferation and survival of mammalian glioma cells [50]. The upregulation of PTPRQ in cells of the TME might therefore induce cell cycle arrest and senescence in these cells, with acquisition of the senescence-associated secretory phenotype (SASP), possibly stimulating cancer cells and increasing the supply of nutrients to the metastasizing cancer cells, which eventually supports cancer cell growth in the brain.

The expressions of* Sspo* and* Pole* were also upregulated in stromal cells of the brain harboring metastases. SCO-spondin is expressed in the subcommissural organ (SCO) and plays a role in neuronal development [51]. It also promotes commissural fiber regrowth and functional recovery after spinal cord injury [52]. The DNA polymerase *ε* catalytic subunit encoded by* Pole* contributes to DNA repair and chromosomal DNA replication [33]. The metastasis of cancer cells to the brain followed by their growth and invasion of surrounding tissue may damage the brain and thereby trigger DNA replication and neuronal regeneration systems. Cancer cells have also been found to release axon guidance molecules and thereby to stimulate the formation of neurites, and they can then exploit the availability of nerve fiber–derived factors that promote cancer cell survival and proliferation [53], suggesting that regeneration of the nervous system is functionally relevant to tumor progression.

The Na^+^-Ca^2+^ exchanger SLC8A2 is restricted to the brain [36], and plasmolipin (PLLP) is restricted to the kidney and myelinated tracts in the brain [54]. However, we have now shown that the expressions of* SLC8A2* and* PLLP* were upregulated in metastasizing breast cancer and melanoma cells in the brain. This finding is consistent with the previous observation that the brain microenvironment induces reprogramming of metastasized cancer cells and their consequent acquisition of neuronal characteristics [18].

GeneMANIA analysis revealed various interactions—including* Tph2*-*CXCR4* and* Sspo*-*TNFSF4*—among the genes whose expression in human metastasizing cancer cells or the mouse brain stroma was associated with metastasis. The serotonin receptor HTR2A is linked to CXCR4, suggesting that serotonin released from reactive astrocytes might affect CXCR4^+^ metastatic cancer cells through this receptor. Further studies are thus warranted to clarify the possible role of serotonin in the regulation of CXCR4 signaling, which plays an important role in brain metastasis. TNFSF4 (OX40L) is expressed on the surface of antigen presenting cells and endothelial cells, and it promotes T cell activation, proliferation, and survival as well as stimulating the antitumor immune response through interaction with its receptor TNFRSF4 (OX40) [55, 56]. OX40L-OX40 signaling also promotes neurogenic inflammation [57], and several agonistic monoclonal antibodies to OX40 are currently being tested in early-phase clinical trials for their efficacy as cancer immunotherapeutic agents [58, 59]. Further studies are necessary to elucidate how the interaction of OX40L-OX40 signaling and SCO-spondin might contribute to the metastasis of cancer cells to the brain.

## 5. Conclusions

Metastasizing cancer cells interact with the tissue microenvironment at the metastatic site. We have now established mouse xenograft models of brain metastasis based on intracardiac injection of human breast cancer or melanoma cell lines to characterize these interactions. RNA-seq analysis of mouse brain tissue harboring human cancer cell metastases identified both mouse and human genes whose expression was specifically associated with metastasis. We focused on the brain microenvironment that contributes to completion of the metastatic process and identified four genes—*Tph2*,* Sspo*,* Ptprq*, and* Pole*—whose expression was specifically upregulated in brain tissue containing metastases. Our approach has thus shed light on the cross talk that is thought to take place between cancer cells and their microenvironment, and it has the potential to provide a basis for the development of novel therapeutic strategies to thwart brain metastasis.

## Supplementary Material

The list of 190 mouse genes that showed statistically significant differences between the meta(+) brains and the control brains.

## Figures and Tables

**Table 1:** The summary of sequence reads from RNA sequencing analysis.

Sample	All reads	Mouse (mm9)		Human (GRCh37)		Mouse + human	
		Reads	Rate (%)	Reads	Rate (%)	Reads	Rate (%)
Control brain #1	45,164,445	41,441,702	91.80%	123,245	0.30%	41,564,947	92.00%
Control brain #2	44,699,341	41,642,344	93.20%	136,200	0.30%	41,778,544	94.00%
231-Luc meta(+) brain #1	45,875,684	39,903,992	87.00%	2,601,796	5.70%	42,505,788	92.70%
231-Luc meta(+) brain #2	47,065,221	37,107,394	78.80%	5,180,347	11.00%	42,287,741	89.80%
Control bone marrow	44,354,308	42,972,236	96.90%	402,488	0.90%	43,374,724	97.80%
231-Luc meta(+) bone marrow	45,782,568	41,998,054	91.70%	1,112,749	2.40%	43,110,803	94.20%
231-Luc fat pad tumor	43,374,724	82,230,795	18.80%	31,246,344	71.40%	39,477,139	90.30%

Data are shown for the brain of control mice injected with PBS (#1, #2), the brain of mice with metastases formed by 231-Luc cells (#1, #2), bone marrow of a control mouse injected with PBS, bone marrow of a mouse injected with 231-Luc cells, and a primary fat pad tumor formed by 231-Luc cells.

## References

[1] Nayak L., Lee E. Q., Wen P. Y. (2012). Epidemiology of brain metastases. *Current Oncology Reports*.

[2] Owonikoko T. K., Arbiser J., Zelnak A. (2014). Current approaches to the treatment of metastatic brain tumours. *Nature Reviews Clinical Oncology*.

[3] Lin X., DeAngelis L. M. (2015). Treatment of brain metastases. *Journal of Clinical Oncology*.

[4] Lai R., Dang C. T., Malkin M. G., Abrey L. E. (2004). The risk of central nervous system metastases after trastuzumab therapy in patients with breast carcinoma. *Cancer*.

[5] Omuro A. M. P., Kris M. G., Miller V. A. (2005). High incidence of disease recurrence in the brain and leptomeninges in patients with nonsmall cell lung carcinoma after response to gefitinib. *Cancer*.

[6] Kikuchi T., Daigo Y., Ishikawa N. (2006). Expression profiles of metastatic brain tumor from lung adenocarcinomas on cDNA microarray. *International Journal of Oncology*.

[7] Saunus J. M., Quinn M. C. J., Patch A.-M. (2015). Integrated genomic and transcriptomic analysis of human brain metastases identifies alterations of potential clinical significance. *Journal of Pathology*.

[8] Bos P. D., Zhang X. H.-F., Nadal C. (2009). Genes that mediate breast cancer metastasis to the brain. *Nature*.

[9] Gaziel-Sovran A., Osman I., Hernando E. (2013). In vivo modeling and molecular characterization: a path toward targeted therapy of melanoma brain metastasis. *Frontiers in Oncology*.

[10] Tominaga N., Kosaka N., Ono M. (2015). Brain metastatic cancer cells release microRNA-181c-containing extracellular vesicles capable of destructing blood-brain barrier. *Nature Communications*.

[11] Hoshino A., Costa-Silva B., Shen T.-L. (2015). Tumour exosome integrins determine organotropic metastasis. *Nature*.

[12] Chen F., Zhuang X., Lin L. (2015). New horizons in tumor microenvironment biology: challenges and opportunities. *BMC Medicine*.

[13] Fidler I. J. (2015). The biology of brain metastasis: challenges for therapy. *The Cancer Journal*.

[14] Obenauf A. C., Massagué J. (2015). Surviving at a distance: organ-specific metastasis. *Trends in Cancer*.

[15] Massagué J., Obenauf A. C. (2016). Metastatic colonization by circulating tumour cells. *Nature*.

[16] Wang H., Yu C., Gao X. (2015). The osteogenic niche promotes early-stage bone colonization of disseminated breast cancer cells. *Cancer Cell*.

[17] Müller A., Homey B., Soto H. (2001). Involvement of chemokine receptors in breast cancer metastasis. *Nature*.

[18] Ren G., Esposito M., Kang Y. (2015). Bone metastasis and the metastatic niche. *Journal of Molecular Medicine*.

[19] Cox T. R., Rumney R. M. H., Schoof E. M. (2015). The hypoxic cancer secretome induces pre-metastatic bone lesions through lysyl oxidase. *Nature*.

[20] Park E. S., Kim S. J., Kim S. W. (2011). Cross-species hybridization of microarrays for studying tumor transcriptome of brain metastasis. *Proceedings of the National Academy of Sciences of the United States of America*.

[21] Conway T., Wazny J., Bromage A. (2012). Xenome—a tool for classifying reads from xenograft samples. *Bioinformatics*.

[22] Raskatov J. A., Nickols N. G., Hargrove A. E., Marinov G. K., Wold B., Dervan P. B. (2012). Gene expression changes in a tumor xenograft by a pyrrole-imidazole polyamide. *Proceedings of the National Academy of Sciences of the United States of America*.

[23] Hosonaga M., Arima Y., Sugihara E., Kohno N., Saya H. (2014). Expression of CD24 is associated with HER2 expression and supports HER2-Akt signaling in HER2-positive breast cancer cells. *Cancer Science*.

[24] Fukuda K., Sugihara E., Ohta S. (2015). Periostin is a key niche component for wound metastasis of melanoma. *PLoS ONE*.

[25] Nori S., Okada Y., Nishimura S. (2015). Long-term safety issues of iPSC-based cell therapy in a spinal cord injury model: oncogenic transformation with epithelial-mesenchymal transition. *Stem Cell Reports*.

[26] Trapnell C., Pachter L., Salzberg S. L. (2009). TopHat: discovering splice junctions with RNA-Seq. *Bioinformatics*.

[27] Liao Y., Smyth G. K., Shi W. (2014). FeatureCounts: an efficient general purpose program for assigning sequence reads to genomic features. *Bioinformatics*.

[28] Robinson M. D., McCarthy D. J., Smyth G. K. (2010). edgeR: a Bioconductor package for differential expression analysis of digital gene expression data. *Bioinformatics*.

[29] Chang H. Y. (2009). Anatomic demarcation of cells: genes to patterns. *Science*.

[30] Walther D. J., Peter J.-U., Bashammakh S. (2003). Synthesis of serotonin by a second tryptophan hydroxylase isoform. *Science*.

[31] Zhang X., Beaulieu J.-M., Sotnikova T. D., Gainetdinov R. R., Caron M. G. (2004). Tryptophan hydroxylase-2 controls brain serotonin synthesis. *Science*.

[32] Gobron S., Creveaux I., Meiniel R. (2000). Subcommissural organ/Reissner's fiber complex: characterization of SCO-spondin, a glycoprotein with potent activity on neurite outgrowth. *GLIA*.

[33] Kesti T., Frantti H., Syvaoja J. E. (1993). Molecular cloning of the cDNA for the catalytic subunit of human DNA polymerase *ε*. *The Journal of Biological Chemistry*.

[34] Hinton C. V., Avraham S., Avraham H. K. (2010). Role of the CXCR4/CXCL12 signaling axis in breast cancer metastasis to the brain. *Clinical and Experimental Metastasis*.

[35] Chen Q., Zhang X. H.-F., Massagué J. (2011). Macrophage binding to receptor VCAM-1 transmits survival signals in breast cancer cells that invade the lungs. *Cancer Cell*.

[36] Lytton J. (2007). Na^+^/Ca^2+^ exchangers: three mammalian gene families control Ca^2+^ transport. *Biochemical Journal*.

[37] Yae T., Tsuchihashi K., Ishimoto T. (2012). Alternative splicing of CD44 mRNA by ESRP1 enhances lung colonization of metastatic cancer cell. *Nature Communications*.

[38] Sun X., Cheng G., Hao M. (2010). CXCL12 / CXCR4 / CXCR7 chemokine axis and cancer progression. *Cancer and Metastasis Reviews*.

[39] Chen Q., Massagué J. (2012). Molecular pathways: VCAM-1 as a potential therapeutic target in metastasis. *Clinical Cancer Research*.

[40] Warde-Farley D., Donaldson S. L., Comes O. (2010). The GeneMANIA prediction server: biological network integration for gene prioritization and predicting gene function. *Nucleic Acids Research*.

[41] Shannon P., Markiel A., Ozier O. (2003). Cytoscape: a software Environment for integrated models of biomolecular interaction networks. *Genome Research*.

[42] Steeg P. S. (2016). Targeting metastasis. *Nature Reviews Cancer*.

[43] Kim S.-J., Kim J.-S., Park E. S. (2011). Astrocytes upregulate survival genes in tumor cells and induce protection from chemotherapy. *Neoplasia*.

[44] Chen Q., Boire A., Jin X. (2016). Carcinoma-astrocyte gap junctions promote brain metastasis by cGAMP transfer. *Nature*.

[45] Valiente M., Obenauf A. C., Jin X. (2014). Serpins promote cancer cell survival and vascular co-option in brain metastasis. *Cell*.

[46] Zhang Y., Chen K., Sloan S. A. (2014). An RNA-sequencing transcriptome and splicing database of glia, neurons, and vascular cells of the cerebral cortex. *The Journal of Neuroscience*.

[47] Sarrouilhe D., Clarhaut J., Defamie N., Mesnil M. (2015). Serotonin and cancer: what is the link?. *Current Molecular Medicine*.

[48] Pai V. P., Marshall A. M., Hernandez L. L., Buckley A. R., Horseman N. D. (2009). Altered serotonin physiology in human breast cancers favors paradoxical growth and cell survival. *Breast Cancer Research*.

[49] Pulido R., Stoker A. W., Hendriks W. J. A. J. (2013). PTPs emerge as PIPs: protein tyrosine phosphatases with lipid-phosphatase activities in human disease. *Human Molecular Genetics*.

[50] Oganesian A., Poot M., Daum G. (2003). Protein tyrosine phosphatase RQ is a phosphatidylinositol phosphatase that can regulate cell survival and proliferation. *Proceedings of the National Academy of Sciences of the United States of America*.

[51] Meiniel A. (2001). SCO-spondin, a glycoprotein of the subcommissural organ/Reissner's fiber complex: evidence of a potent activity on neuronal development in primary cell cultures. *Microscopy Research and Technique*.

[52] Sakka L., Delétage N., Lalloué F. (2014). SCO-spondin derived peptide NX210 induces neuroprotection in vitro and promotes fiber regrowth and functional recovery after spinal cord injury. *PLoS ONE*.

[53] Mancino M., Ametller E., Gascón P., Almendro V. (2011). The neuronal influence on tumor progression. *Biochimica et Biophysica Acta*.

[54] Cochary E. F., Bizzozero O. A., Sapirstein V. S., Nolan C. E., Fischer I. (1990). Presence of the plasma membrane proteolipid (plasmolipin) in myelin. *Journal of Neurochemistry*.

[55] Pardoll D. M. (2012). The blockade of immune checkpoints in cancer immunotherapy. *Nature Reviews Cancer*.

[56] Redmond W. L., Ruby C. E., Weinberg A. D. (2009). The role of OX40-mediated co-stimulation in T-cell activation and survival. *Critical Reviews in Immunology*.

[57] Sonar S., Lal G. (2015). Role of tumor necrosis factor superfamily in neuroinflammation and autoimmunity. *Frontiers in Immunology*.

[58] Curti B. D., Kovacsovics-Bankowski M., Morris N. (2013). OX40 is a potent immune-stimulating target in late-stage cancer patients. *Cancer Research*.

[59] Aspeslagh S., Postel-Vinay S., Rusakiewicz S., Soria J.-C., Zitvogel L., Marabelle A. (2016). Rationale for anti-OX40 cancer immunotherapy. *European Journal of Cancer*.

