# Supplementary material for: RNA Sequencing Analysis Reveals Interactions between Breast Cancer or Melanoma Cells and the Tissue Microenvironment during Brain Metastasis
Source: Biomed Res Int. 2017 Jan 22;2017:8032910. doi: 10.1155/2017/8032910 (PMC5292181; doi:10.1155/2017/8032910)
Supplement: Supplementary file 1 — The list of 190 mouse genes that showed statistically significant differences between the meta(+) brains and the control brains. [file 8032910.f1.docx]

**Supporting Information**

**Supplemental Table 1.** Genes whose expression was found to be significantly up- or down-regulated in the brain of mice with metastases relative to that in the control brain. Shades of red shows the up-regulated genes.

| Gene | log fold change (FC) | log counts per million reads (CPM) | *P*-value |
| --- | --- | --- | --- |
| Col5a3 | 2.363590911 | 4.04038848 | 0.001638687 |
| Ano3 | -1.653624303 | 5.31214368 | 0.002211247 |
| Dio2 | -1.530935622 | 6.662820298 | 0.004725779 |
| Tgm1 | 6.089639078 | 2.63470163 | 0.007920543 |
| Aspg | 1.978751267 | 4.037382621 | 0.009080364 |
| Exd2 | -1.277228832 | 5.778826595 | 0.010340404 |
| Nes | 2.145008594 | 4.326802557 | 0.013293763 |
| Gpr88 | -2.272837142 | 6.960524409 | 0.015481448 |
| Myo15 | 5.295643885 | 3.416710533 | 0.018022383 |
| Tomm40l | -1.181313538 | 5.848569206 | 0.020441614 |
| Lmbrd2 | -1.066162435 | 4.836740313 | 0.02179239 |
| Oasl2 | 2.771242497 | 3.465777356 | 0.022574512 |
| Gm3362 | -1.692602804 | 4.269223536 | 0.023380343 |
| Shc3 | -1.433909556 | 3.884735303 | 0.024244304 |
| Ctla2a | 1.879168527 | 4.231823617 | 0.024453782 |
| Etohi1 | -1.033266421 | 5.434699304 | 0.025059664 |
| Cp | 1.524811698 | 5.467947706 | 0.02596001 |
| Rph3a | 3.092360498 | 5.660228044 | 0.026100199 |
| Neb | 3.864396119 | 4.435552089 | 0.026369066 |
| Mvp | 1.526622222 | 4.202417798 | 0.026899545 |
| Tph2 | 3.069117774 | 4.430399867 | 0.02762446 |
| Dlx6os1 | -1.520354175 | 8.19711298 | 0.027661361 |
| Dlx6os1 | -1.277698331 | 4.179438177 | 0.029530118 |
| Coch | -1.163485539 | 4.843971845 | 0.030003337 |
| Sspo | 3.311372872 | 3.997700447 | 0.030432011 |
| Fnbp1l | -1.260220193 | 6.883938639 | 0.030950838 |
| Niacr1 | 6.529042834 | 1.639405498 | 0.032845695 |
| Rlbp1 | -1.320968006 | 3.746655726 | 0.032941708 |
| Dlx6os1 | -1.562061514 | 3.921656037 | 0.033687792 |
| Dnahc11 | 3.650280694 | 3.853594885 | 0.035996438 |
| Serpina3n | 2.569265498 | 7.463363537 | 0.036132147 |
| Khdrbs3 | -1.252174019 | 6.153135808 | 0.036343237 |
| Gp49a | 5.449408162 | 1.754892645 | 0.036886435 |
| Nr1h4 | 4.686603757 | 2.029400551 | 0.0370231 |
| Lgals3bp | 1.424886179 | 4.903230709 | 0.03709184 |
| Kctd4 | -1.056737902 | 5.013772926 | 0.038537064 |
| Otog | 6.751419709 | 3.106999295 | 0.039128538 |
| Zmym1 | -1.092551963 | 4.840488271 | 0.040501437 |
| Fam118b | -0.932000417 | 4.629187374 | 0.040792562 |
| Zan | 4.373945737 | 3.867994202 | 0.042234505 |
| Tmed5 | -0.836444693 | 5.21237008 | 0.042318671 |
| Tmem181a | -1.107308525 | 5.452974509 | 0.044608166 |
| Nat14 | -0.942221988 | 5.004598859 | 0.044773376 |
| Mylk4 | 5.766647916 | 2.406657809 | 0.044869069 |
| Ptprq | 6.288694235 | 2.649569524 | 0.046100806 |
| Sis | 5.458116324 | 2.487706811 | 0.046929291 |
| Myo7b | 8.303713148 | 2.539577222 | 0.048011063 |
| Lrrc10b | -1.880687639 | 3.799372837 | 0.04832796 |
| Ovgp1 | 1.438227943 | 3.864849996 | 0.048470888 |
| Dysf | 2.013415161 | 3.730051739 | 0.048540616 |
| Alpk3 | 3.862344922 | 2.889922951 | 0.049004807 |
| Pole | 3.123970251 | 3.088452701 | 0.049341058 |
| Dnahc17 | 3.663820327 | 3.808204578 | 0.049517917 |
| Grm3 | -1.539813595 | 5.385632929 | 0.00036613 |
| Egr3 | -1.734704753 | 3.926251096 | 0.005329548 |
| Cd109 | 2.753468852 | 3.776185107 | 0.006651788 |
| Lgals3 | 3.153625803 | 3.572427097 | 0.008017502 |
| Lox | 3.465480201 | 3.291162006 | 0.014953585 |
| Ccl2 | 5.391661754 | 2.329312299 | 0.020188581 |
| Cxcl10 | 6.827643641 | 1.827129231 | 0.028014334 |
| Gng7 | -1.839537587 | 7.602111494 | 0.028273765 |
| Mmp3 | 7.381652348 | 1.603150295 | 0.030475785 |
| Ccl6 | 3.455104072 | 3.132368368 | 0.032219559 |
| Icam1 | 2.561141578 | 3.198160901 | 0.034825098 |
| Cd44 | 1.577361215 | 4.161911152 | 0.041069029 |
| Gm14326 | -2.113452664 | 5.167161933 | 6.44E-07 |
| Gm14325 | -1.963542538 | 4.887392906 | 1.39E-06 |
| OTTMUSG00000016609 | -2.264905926 | 5.112125507 | 2.16E-05 |
| 0610010B08Rik | -2.277064373 | 4.725109651 | 0.000148274 |
| Gm14430 | -2.293831333 | 4.755578297 | 0.000247564 |
| Gm14434 | -2.274455121 | 4.757872838 | 0.000265909 |
| Gm6710 | -2.116683142 | 4.239961731 | 0.000684492 |
| C030023E24Rik | -1.488128979 | 5.038806458 | 0.002658482 |
| 6530418L21Rik | -1.349377806 | 5.293008889 | 0.009794022 |
| Gm14391 | -1.147663098 | 4.387868898 | 0.01343878 |
| 100043387 | -2.351282028 | 3.045587907 | 0.014803868 |
| Gm14308 | -2.313605839 | 2.885110496 | 0.024791228 |
| LOC100504405 | -1.809443345 | 4.329825087 | 0.028139436 |
| Gm8759 | -1.844137383 | 4.023924167 | 0.028350182 |
| Gm12191 | -1.492522767 | 4.58312793 | 0.028562247 |
| 2810408P10Rik | -1.041684785 | 4.556866545 | 0.02908841 |
| LOC100504012 | -1.34726436 | 3.918185051 | 0.030239981 |
| Gm14288 | -1.127279367 | 5.672770772 | 0.032036683 |
| D430019H16Rik | -1.330712056 | 3.913668074 | 0.036490268 |
| Gm5878 | 10.74005931 | 2.078629207 | 0.038839708 |
| Gm14420 | -0.938503134 | 5.536630678 | 0.038966185 |
| 5730590G19Rik | 5.855690056 | 2.705515598 | 0.039555778 |
| 4932431P20Rik | 8.685255697 | 2.852452069 | 0.04004586 |
| Gm14322 | -1.016560541 | 4.240646691 | 0.047101861 |
| Gm10191 | -1.937248756 | 3.859853514 | 0.047518495 |
| 2810021G02Rik | -1.483490143 | 3.337872762 | 0.0481903 |
| 1190002H23Rik | -1.084866859 | 4.043938943 | 0.048678157 |
| Gm10132 | -1.268402308 | 4.302677356 | 0.048939245 |
| C920009B18Rik | 10.70186761 | 2.044252772 | 0.049154135 |
| Osmr | 2.975562404 | 4.322592295 | 0.000102764 |
| Saa3 | 8.611301385 | 2.705031185 | 0.000477577 |
| Kcnv1 | -1.827224154 | 5.518999553 | 0.002355439 |
| Plin4 | 2.29219774 | 5.385595117 | 0.00412951 |
| C4a | 1.746228843 | 4.74319856 | 0.004289224 |
| Avp | -3.415214581 | 3.204052701 | 0.004364854 |
| Mfge8 | -1.707886263 | 6.66905029 | 0.004591572 |
| Gpr116 | 1.825479255 | 4.406930874 | 0.005191999 |
| Homer1 | -1.576409891 | 6.6634521 | 0.005277761 |
| Dgkb | -1.622770447 | 6.80621212 | 0.00534543 |
| A2m | 2.654195423 | 5.838701921 | 0.005563245 |
| Ly6a | 2.992800685 | 4.108127633 | 0.005613943 |
| Timp1 | 4.164451147 | 3.869855125 | 0.005690731 |
| Lix1 | -1.382409872 | 5.850606173 | 0.005944557 |
| Vwf | 2.131330503 | 4.341096984 | 0.007018024 |
| Socs3 | 2.63731838 | 3.840469787 | 0.007049866 |
| Hmox1 | 3.443693713 | 5.567381551 | 0.007116442 |
| Lcn2 | 5.202814363 | 4.75857513 | 0.008121851 |
| Cdr1 | -1.345415298 | 5.304705682 | 0.00873552 |
| Cyr61 | 2.595270852 | 4.771302204 | 0.008998446 |
| Fcgr2b | 2.249569518 | 4.278648288 | 0.010278619 |
| Rcn1 | -1.134519696 | 4.946754194 | 0.010299944 |
| Flnc | 2.587636535 | 4.073870501 | 0.011243284 |
| Nptx2 | -1.302911771 | 4.189073331 | 0.011563761 |
| S1pr3 | 2.147120412 | 4.219875367 | 0.012542325 |
| Lilrb4 | 4.509208917 | 2.52075172 | 0.012593128 |
| Apob | 6.332549988 | 3.605130678 | 0.013328965 |
| P2ry12 | -1.269262557 | 5.367492902 | 0.015323263 |
| Adamts1 | 1.413018911 | 4.743895848 | 0.0153593 |
| Lrrk2 | -1.222649023 | 5.448226151 | 0.015883912 |
| H19 | 3.812479962 | 3.444378624 | 0.015999161 |
| Sbno2 | 1.238219247 | 4.674771428 | 0.016476033 |
| Fsip2 | 12.90279529 | 4.10969182 | 0.017229538 |
| Rims1 | -1.377612186 | 6.323575255 | 0.018369291 |
| Ttn | 5.680684711 | 6.433539056 | 0.019588839 |
| Hspg2 | 2.140519128 | 4.46980484 | 0.020076061 |
| Ifit1 | 3.104651104 | 3.155390423 | 0.020156416 |
| Gpr17 | -1.177705163 | 5.107236334 | 0.020295107 |
| Lrp2 | 4.357746966 | 3.839998231 | 0.020357123 |
| Msr1 | 4.058608955 | 2.986336325 | 0.020388221 |
| Csf2rb | 3.022858797 | 3.140556403 | 0.021162904 |
| Ifit3 | 2.143228442 | 3.843559016 | 0.021279869 |
| C3 | 3.219135757 | 3.114325812 | 0.021307345 |
| Camkk2 | -1.333157019 | 5.880294153 | 0.021548641 |
| Ifitm3 | 2.024971278 | 4.471637053 | 0.022002926 |
| Ephx2 | -1.181456417 | 4.094088606 | 0.023643862 |
| Ubxn2b | -1.00767755 | 4.859464817 | 0.024024519 |
| Mybpc1 | 2.72714897 | 4.348711319 | 0.024200779 |
| Lama5 | 1.962536048 | 4.260834317 | 0.024566559 |
| Pak3 | -1.099657888 | 4.410560996 | 0.024633927 |
| Plce1 | 1.703631913 | 6.018163263 | 0.024911424 |
| Gpnmb | 3.97998259 | 2.836280244 | 0.025249518 |
| Eppk1 | 5.081992057 | 3.374586082 | 0.025558638 |
| Thbs1 | 2.355784275 | 3.682144157 | 0.025611122 |
| Slc43a3 | 3.429514642 | 2.927786982 | 0.026701143 |
| Runx3 | 10.57518767 | 1.920114196 | 0.026926218 |
| Spp1 | 2.958995958 | 6.025086922 | 0.027777674 |
| Lpl | -1.160207543 | 5.295997421 | 0.028182414 |
| Cd93 | 2.334777328 | 3.574396854 | 0.028766741 |
| Ier5 | -0.97913745 | 5.269498378 | 0.030267365 |
| P4ha1 | -0.911810577 | 5.344201823 | 0.030472528 |
| Cngb3 | 10.77521737 | 2.108382871 | 0.030765139 |
| Ush2a | 6.114177568 | 3.746435995 | 0.031378134 |
| Pcsk1 | -0.944956807 | 4.515438035 | 0.031635288 |
| H3f3a | -1.355112556 | 5.25246918 | 0.032030577 |
| Serpina3m | 2.808830512 | 3.253972809 | 0.032848323 |
| Ncam2 | -0.942995307 | 5.533395293 | 0.033358182 |
| Tmem119 | -0.963964904 | 4.37340652 | 0.033742669 |
| C4b | 1.860285631 | 6.91057022 | 0.03379496 |
| Gldc | -0.888108711 | 4.60898563 | 0.033897033 |
| Rgs20 | -1.114846638 | 4.487180499 | 0.034520041 |
| Slc2a4 | 2.675819026 | 3.014372402 | 0.034526345 |
| Ctgf | 1.401069581 | 4.759081532 | 0.0353531 |
| Col4a6 | 6.010393919 | 2.611218968 | 0.035920814 |
| Cebpd | 1.969108752 | 3.79961395 | 0.036205908 |
| Siglec1 | 3.767487485 | 2.984168545 | 0.036209337 |
| Tg | 7.278867828 | 2.886878397 | 0.036677832 |
| Ptprb | 1.348259009 | 4.739269004 | 0.037892721 |
| Kcnt2 | -1.188157823 | 4.199270319 | 0.038164146 |
| Muc6 | 5.187349399 | 2.892490008 | 0.03828327 |
| Tenc1 | 1.014146547 | 4.846027669 | 0.039552383 |
| Map3k6 | 2.272226159 | 3.457545977 | 0.039606547 |
| Dnahc10 | 2.949201402 | 3.90269739 | 0.040273826 |
| Fbln5 | 1.738370922 | 4.104169462 | 0.040608827 |
| Cybb | 3.02041808 | 3.029582318 | 0.041049845 |
| Mgam | 11.21555806 | 2.511731646 | 0.042031634 |
| Osbpl8 | -1.158082147 | 6.923877544 | 0.042170591 |
| Nlrc5 | 4.114520768 | 2.866238268 | 0.042649205 |
| Muc4 | 12.00186652 | 3.246438672 | 0.043300512 |
| Aspm | 4.405030465 | 3.192798583 | 0.044242316 |
| Tnfrsf19 | -1.020302808 | 5.940952526 | 0.044915922 |
| Vim | 2.329696091 | 7.445618786 | 0.045199279 |
| Grm5 | -1.203338865 | 7.592165938 | 0.045493171 |
| Prkar2b | -1.096933851 | 6.125468424 | 0.046480484 |
| Nipal1 | 7.708271261 | 1.992386025 | 0.046682806 |
| Pde10a | -1.648337017 | 7.19797807 | 0.047266739 |
| Pex6 | -0.869341352 | 5.189464445 | 0.048564051 |
| Lhx2 | -0.979902794 | 4.990581375 | 0.049250493 |
| Parp14 | 2.729393467 | 3.265594034 | 0.049968173 |
